# GLUT3 and PKM2 regulate OCT4 expression and support the hypoxic culture of human embryonic stem cells

**DOI:** 10.1038/srep17500

**Published:** 2015-12-07

**Authors:** David R. Christensen, Philip C. Calder, Franchesca D. Houghton

**Affiliations:** 1 Centre for Human Development, Stem Cells & Regeneration, Faculty of Medicine, University of Southampton, Southampton, SO16 6YD, UK; 2 Human Development and Health Academic Unit, Faculty of Medicine, University of Southampton, Southampton, SO16 6YD, UK; 3 NIHR Southampton Biomedical Research Centre, University of Southampton and University Hospital Southampton NHS Foundation Trust, Southampton, SO16 6YD, UK

## Abstract

Human embryonic stem cells (hESCs) have the capacity to differentiate into all cell types and thus have great potential for regenerative medicine. hESCs cultured at low oxygen tensions are more pluripotent and display an increased glycolytic rate but how this is regulated is unknown. This study therefore aimed to investigate the regulation of glucose metabolism in hESCs and whether this might impact OCT4 expression. In contrast to the glucose transporter GLUT1, GLUT3 was regulated by environmental oxygen and localised to hESC membranes. Silencing GLUT3 caused a reduction in glucose uptake and lactate production as well as OCT4 expression. GLUT3 and OCT4 expression were correlated suggesting that hESC self-renewal is regulated by the rate of glucose uptake. Surprisingly, PKM2, a rate limiting enzyme of glycolysis displayed a nuclear localisation in hESCs and silencing PKM2 did not alter glucose metabolism suggesting a role other than as a glycolytic enzyme. PKM2 expression was increased in hESCs cultured at 5% oxygen compared to 20% oxygen and silencing PKM2 reduced OCT4 expression highlighting a transcriptional role for PKM2 in hESCs. Together, these data demonstrate two separate mechanisms by which genes regulating glucose uptake and metabolism are involved in the hypoxic support of pluripotency in hESCs.

Human embryonic stem cells (hESCs) are derived from the inner cell mass of the blastocyst and are pluripotent; they have the capacity to differentiate into all cell-types in the human body^1^,^2^,^3^,^4^. Thus hESCs have great potential to provide cellular therapy for a range of diseases. For this hope to be realised with safety and efficiency, hESCs need to be maintained as highly pluripotent populations in the absence of spontaneous differentiation. Much data suggests that environmental culture conditions and specifically the oxygen tension have an impact on the maintenance of pluripotency.

Use of low environmental oxygen tensions has been shown to reduce the amount of spontaneous differentiation, as well as being beneficial for hESC maintenance in terms of increased expression of key pluripotency markers and decreased incidence of chromosomal abnormalities^5^,^6^,^7^,^8^,^9^,^10^. Maintenance of hESCs at atmospheric oxygen has also been found to decrease hESC proliferation and glycolytic and amino acid metabolism of hESCs^9^,^10^,^11^. Higher rates of glucose uptake and lactate production were measured in hESCs cultured at 5% oxygen than in those maintained at 20% oxygen, which was mirrored by the increased expression of the pluripotency markers OCT4, SOX2, and NANOG^9^. Interestingly, this association between glycolytic metabolism and pluripotency was also demonstrated in hESCs cultured at 5% oxygen in the absence of FGF2, where a reduction of SOX2 expression, glucose uptake and lactate production was observed when compared with hESCs cultured in the presence of FGF2^9^. These findings suggest that a high rate of glucose uptake and lactate production is characteristic of highly pluripotent stem cells and that hypoxia might be beneficial for the maintenance of hESCs at least partially by supporting glycolytic metabolism. Importantly, expression of many glycolytic genes has been shown to be promoted under hypoxia in other cell-types, providing a mechanism by which hypoxic conditions might regulate metabolism in hESCs^12^,^13^,^14^,^15^,^16^.

How glucose metabolism is regulated in hESCs is not known, but entry into the cell via glucose transporters is likely to be key. However, which glucose transporter is responsible for glucose uptake in hESCs is not known. The glucose transporter GLUT1 has been found in many cell types, and its expression has been found to be regulated by hypoxia in mouse ESCs (mESCs)^17^,^18^,^19^,^20^. Expression of *GLUT1* mRNA was also found to be increased in hESCs cultured at 5% oxygen compared with those at atmospheric oxygen, suggesting that its regulation may drive changes in rates of glucose consumption with changing environmental oxygen tension^9^. This hypoxic promotion of GLUT1 expression was demonstrated to be regulated by HIF-2α^9^. GLUT3 had been considered to be a neuron-specific glucose transporter, but a much wider tissue distribution has since been demonstrated in humans^21^,^22^,^23^. GLUT3 has a higher affinity for glucose than GLUT1 and has a high turnover, which makes it an efficient transporter^24^,^25^. Silencing GLUT3 expression in murine blastocysts led to a greater decrease in glucose uptake than silencing GLUT1 expression, suggesting that GLUT3 might be more important for glucose uptake, at least in preimplantation development^26^. Expression of both transporters, GLUT1 and GLUT3, is regulated by hypoxia in mouse blastocysts^27^.

Glucose utilisation may also be regulated through the activity of glycolytic enzymes. Pyruvate kinase catalyses the breakdown of phosphoenolpyruvate to produce pyruvate and ATP. As this reaction is the final rate-limiting step of glycolysis, it is possible that the rate of glucose uptake and lactate production is controlled through regulation of this step. PKM1 and PKM2 are two splice variants of the *PKM2* gene that differ by only 23 amino acids due to alternatively spliced exons 9 or 10, respectively^28^. PKM2 has been found to promote the Warburg effect in cancer cells, which describes an increased reliance on glycolysis even when enough oxygen is available for oxidative phosphorylation^29^,^30^,^31^. Knockdown of PKM2 in cancer cell lines resulted in decreased rates of glycolytic metabolism and reduced cell viability, but, interestingly, cell viability was not reduced after PKM2 knockdown in human adult skin fibroblasts or human umbilical vein endothelial cells^29^,^32^. It may be that non-catalytic roles for PKM2 explain this difference between cancerous and non-cancerous cells as PKM2 has been found to have roles in transcriptional regulation that include cooperative interactions with OCT4 and HIF-1α^33^,^34^,^35^. Although PKM2 has been shown to be highly expressed in pluripotent stem cells^36^, whether it is involved in the regulation of transcription in hESCs is unknown. Interestingly, one recent paper demonstrated that expression of Pkm2 and Hk2 in mESCs is transcriptionally regulated by Oct4, highlighting an additional mechanism by which glycolytic metabolism might be supported in hESCs^37^. That same study also showed that overexpression of Pkm2 and Hk2 in mESCs was able to support pluripotency in the absence of LIF, which suggested the importance of glycolytic metabolism for maintenance of pluripotency.

This study aims to investigate how glucose metabolism is regulated and how this might impact hESC pluripotency. GLUT3 was demonstrated to be responsible for glucose uptake in hESCs and its expression was found to be up-regulated under hypoxic conditions. Importantly, silencing expression of GLUT3 was shown to decrease glucose consumption and lactate production and lead to a loss of pluripotency. In addition, PKM2 expression was also shown to be up-regulated under hypoxia and was found to be localised to the nucleus of hESCs suggesting a role other than as a glycolytic enzyme. Instead, PKM2 was found to regulate OCT4 expression. These data demonstrate two mechanisms by which a hypoxic environment can be beneficial to hESC pluripotency; promotion of glucose uptake and glycolysis, and the regulation of PKM2 expression, which has a transcriptional role in hESCs.

## Results

### GLUT3 is localised to the membrane of hESCs and is regulated by environmental oxygen

**Figure 1:**
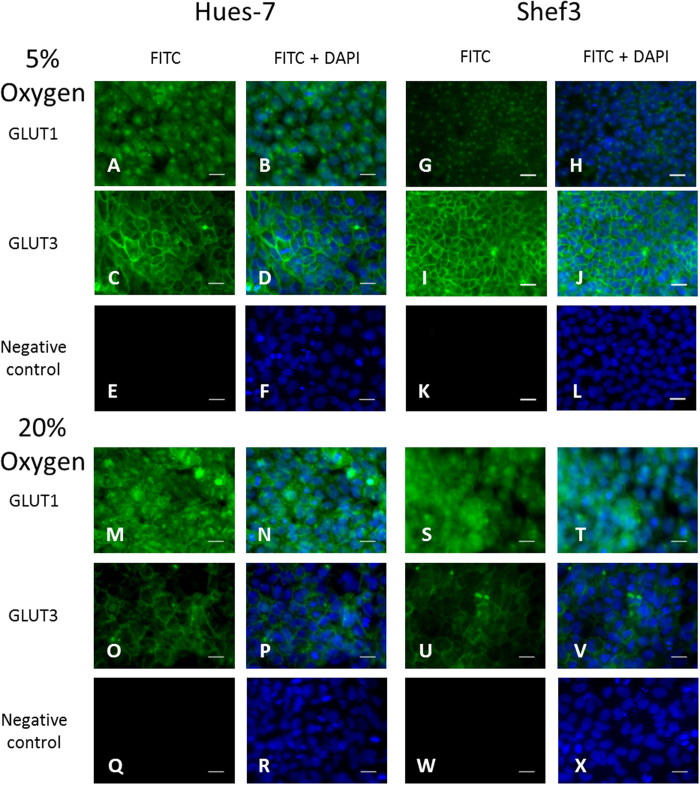
GLUT3 is localised to the membrane of hESCs cultured at either 5% or 20% oxygen. Hues-7 and Shef3 hESCs cultured on a MEF feeder layer at either 5% or 20% oxygen were labelled for GLUT1 (**A,B,G,H,M,N,S,T**) or GLUT3 (**C,D,I,J,O,P,U,V**). FITC-tagged secondary antibodies were used for each protein of interest and for negative, secondary antibody only controls (**E,F,K,L,Q,R,W,X**). DAPI was used to visualise the nuclei (**B,D,F,H,J,L,N,P,R,T,V,X**). GLUT1 expression was not detected at the membrane in either cell-line at either oxygen concentration (**A,G,M,S**). In contrast, GLUT3 expression was detected in the membrane of Hues-7 and Shef3 hESCs at 5% and 20% oxygen (**C,I,O,U**). Scale bar = 25 μm.

hESCs cultured at 5% and 20% oxygen have previously been demonstrated to rely on glucose uptake and on glycolytic metabolism for energy generation^9^. The current study aimed to investigate the control of glycolysis in hESCs and to determine which glucose transporter was responsible for glucose uptake. Immunocytochemistry was used to investigate the localisation of GLUT1 and GLUT3 in two hESC lines maintained at either 5% or 20% oxygen (Fig. 1). GLUT1 was expressed in the cytoplasm in Hues-7 (Fig. 1A,B,M,N) and Shef3 (Fig. 1G,H,S,T) hESCs cultured at either 5% or 20% oxygen. In contrast, GLUT3 expression was detected in the membranes of Hues-7 (Fig. 1C,D,O,P) and Shef3 (Fig. 1I,J,U,V) hESCs cultured at either oxygen tension.

**Figure 2:**
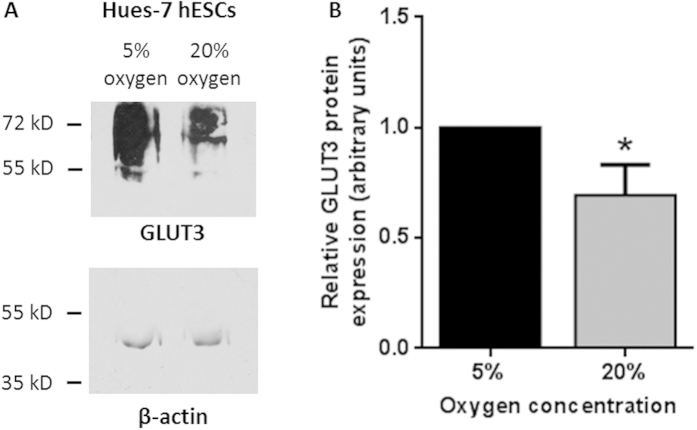
GLUT3 expression is greater in hESCs cultured under hypoxia than in those maintained at atmospheric oxygen. Representative Western blot (**A**) used to quantify GLUT3 protein expression in Hues-7 hESCs cultured at either 5% or 20% oxygen using the housekeeping protein β-actin as a loading control. GLUT3 expression was calculated relative to β-actin expression and expression at 20% oxygen was measured relative to the expression level in hESCs cultured at 5% oxygen. (**B**) A significantly lower expression of GLUT3 was measured in Hues-7 hESCs cultured at 20% compared with those at 5% oxygen (n = 8; p = 0.0443). Bars represent mean ± s.e.m.

Qualitatively, it appeared that there was a reduced level of expression of GLUT3 in hESCs cultured at 20% oxygen in comparison with those maintained at 5% oxygen (Fig. 1). To provide a quantitative measure, Western blotting was used to compare GLUT3 expression in hESCs cultured at either 5% or 20% oxygen (Fig. 2). GLUT3 expression was significantly reduced in Hues-7 hESCs cultured at 20% oxygen compared with those cultured under hypoxic conditions (p = 0.0443; Fig. 2B).

### GLUT3 is responsible for glucose uptake, and regulates glucose metabolism and hESC pluripotency

**Figure 3:**
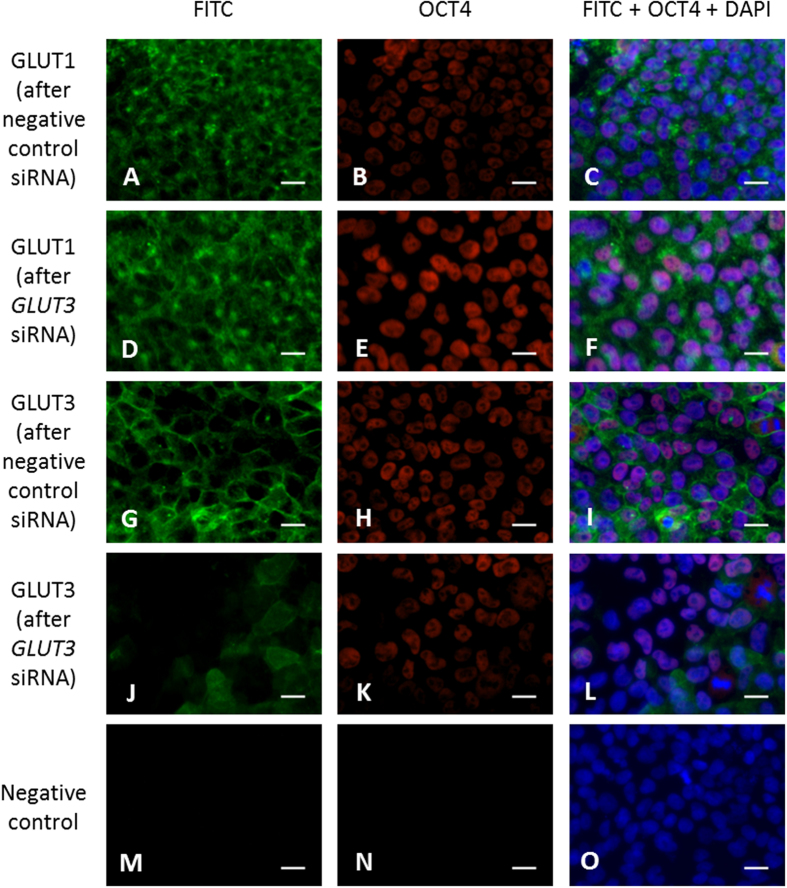
GLUT3 expression is reduced in hESCs after transfection with GLUT3 siRNA. Hues-7 hESCs cultured feeder-free at 5% oxygen were labelled for GLUT1 (**A**,**D**) or GLUT3 (**G**,**J**), and dual-labelled for OCT4 (**B,E,H,K,N**), after transfection with either a negative control siRNA or GLUT3 siRNA. A FITC-tagged secondary antibody was used for GLUT1 and GLUT3 antibodies and a Cy3-tagged secondary antibody was used for OCT4. Both were used for negative, secondary only controls (**M,N**). DAPI was used to visualise the nuclei (**C,F,I,L,O**). A reduction in expression of GLUT3 was seen after transfection with GLUT3 siRNA (**J**), whereas there was no effect on GLUT1 expression (**D**). OCT4 expression was detected after transfection with negative control siRNA and after transfection with GLUT3 siRNA (**B,E,H,K,N**). Scale bar = 25 μm.

siRNA was used to investigate the effect of silencing GLUT3 on hESC metabolism and pluripotency. Hues-7 hESCs, maintained at 5% oxygen, were transfected with 50 nM GLUT3 siRNA. Immunocytochemistry was performed to assess the effect of silencing GLUT3 on the GLUT1 and GLUT3 protein expression in Hues-7 hESCs cultured at 5% oxygen (Fig. 3). The hESCs were dual-labelled with an antibody against OCT4 to qualitatively determine whether silencing GLUT3 expression affects pluripotency. A membranous localisation of GLUT3 was observed for Hues-7 hESCs transfected with a negative control siRNA (Fig. 3G). However, when GLUT3 was silenced, a reduced expression of GLUT3 was observed at the membrane (Fig. 3J). OCT4 was expressed in Hues-7 hESCs maintained at 5% oxygen with or without transfection of siRNA against *GLUT3* (Fig. 3B,E,H,K). As immunocytochemistry is not a quantitative technique, care should be taken when interpreting apparent differences in the expression level of OCT4 in Fig. 3. A more quantitative method, such as Western blotting should be performed to determine whether silencing GLUT3 expression affects expression of OCT4. The level and localisation of GLUT1 expression was not affected following transfection with siRNA against *GLUT3* (Fig. 3A,D). This suggests that GLUT1 was not able to compensate for the lack of GLUT3, as it cannot regulate glucose uptake if it is not localised to the cell membrane.

**Figure 4:**
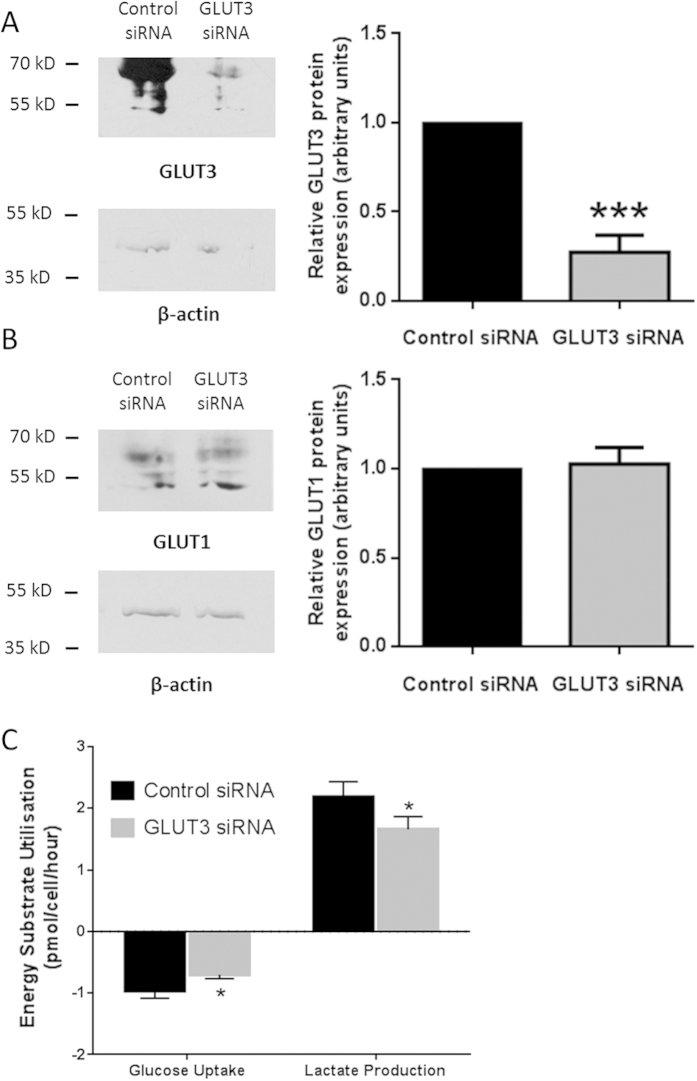
Silencing GLUT3 expression in hESCs reduces the rates of glucose uptake and lactate production without any effect on GLUT1 expression. Hues-7 hESCs were cultured at 5% oxygen and transfected with either a negative control siRNA or GLUT3 siRNA. Western blotting was used to quantify protein expression for GLUT3 (**A**) and GLUT1 (**B**) after transfection. Representative blots for GLUT3, GLUT1, and β-actin expression are shown. Data were normalised to β-actin and to 1 for control siRNA-treated cells. (**A**) A significantly lower level of expression of GLUT3 was measured in Hues-7 hESCs after transfection with GLUT3 siRNA, compared with those transfected with a negative control siRNA (p < 0.001; n = 8). (**B**) No significant difference was found for GLUT1 expression in Hues-7 hESCs transfected with GLUT3 siRNA or negative control siRNA (n = 3). (**C**) The rates of glucose uptake and lactate production were significantly lower in Hues-7 hESCs cultured at 5% oxygen and transfected with GLUT3 siRNA (n = 42 and n = 36, respectively) than in those transfected with a negative control siRNA (n = 43 and n = 40, respectively; p = 0.0347 and p = 0.0435, respectively). Bars represent mean ± s.e.m.

The effect of transfection with siRNA against *GLUT3* on GLUT3 expression was quantitatively measured using Western blotting (Fig. 4A). An approximate 70% reduction in protein expression was measured in comparison with that measured in hESCs transfected with a negative control siRNA (Fig. 4A; p < 0.001). In contrast, silencing GLUT3 did not affect GLUT1 expression (Fig. 4B).

To determine whether silencing GLUT3 altered glucose metabolism, enzyme-linked assays were used to investigate the rate of glucose consumption and lactate production by Hues-7 hESCs cultured at 5% oxygen. The rates of glucose depletion from, and production of lactate into, the medium by Hues-7 hESCs cultured at 5% were determined, following a 2 hour incubation. The rate of glucose depletion was significantly reduced from 0.96 ± 0.12 pmol/cell/hour to 0.70 ± 0.06 pmol/cell/hour after silencing GLUT3 (Fig. 4C; p = 0.0347). Similarly, there was a significant, approximate 25%, reduction in the rate of lactate production (Fig. 4C; p = 0.0435).

**Figure 5:**
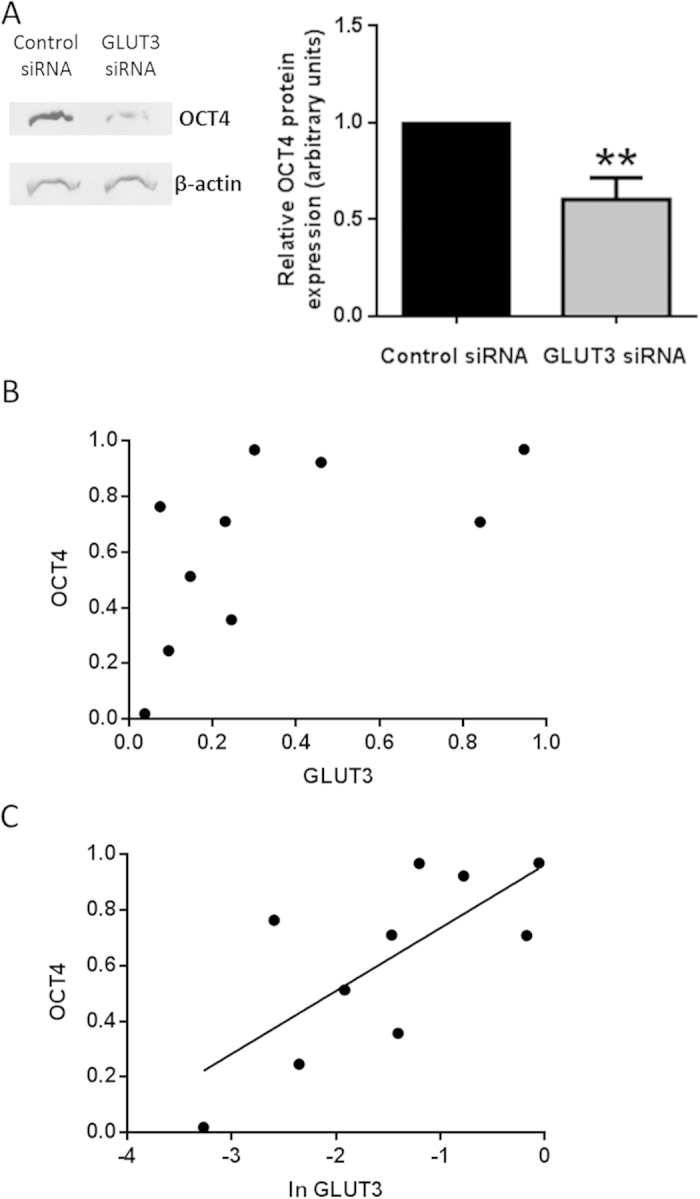
OCT4 and GLUT3 expression are positively correlated. Hues-7 hESCs were cultured at 5% oxygen and transfected with either a negative control siRNA or GLUT3 siRNA. (**A**) Western blotting was used to quantify OCT4 protein expression after transfection. Representative blots for OCT4 and β-actin are shown. Data were normalised to β-actin and to 1 for control siRNA-treated cells. A significantly lower level of expression of OCT4 was measured in Hues-7 hESCs after transfection with GLUT3 siRNA, compared with those transfected with a negative control siRNA (p = 0.0038; n = 8). Bars represent mean ± s. e. m. (**B**) GLUT3 and OCT4 protein expression were quantified in Hues-7 hESCs that were maintained at 20% oxygen, 5% oxygen, 5% oxygen and transfected with GLUT3 siRNA, or 5% oxygen and transfected with a negative control siRNA. The expression level of OCT4 and GLUT3 was calculated for hESCs maintained at 20% oxygen relative to that measured in 5% oxygen, or for hESCs maintained at 5% oxygen and transfected with GLUT3 relative to that measured after transfection with a negative control siRNA. Relative OCT4 expression was plotted against relative GLUT3 expression in the same samples to investigate the relationship between GLUT3 and OCT4. There was a statistically significant positive correlation between OCT4 and GLUT3 expression (p = 0.0438; r = 0.6606). (**C**) The relative measures of expression of GLUT3 were log transformed and plotted against measures of relative OCT4 expression. A statistically significant positive correlation was found between OCT4 and log transformed GLUT3 (p = 0.0185; r = 0.7217).

Western blotting was also used to quantitatively investigate the effect of silencing GLUT3 on OCT4 expression and demonstrated a significant reduction in expression (Fig. 5A; p = 0.0038). To determine whether the expression of GLUT3 was correlated with that of OCT4, both proteins were quantified in Hues-7 hESCs cultured at 5% or 20% oxygen using Western blotting. Band intensity data were normalised to β-actin and to 1 for 5% oxygen, to determine the expression of OCT4 and GLUT3 in hESCs maintained at 20% oxygen relative to the expression level of these proteins in hESCs cultured at 5% oxygen. For the purpose of correlation, the relative expression of OCT4 in Hues-7 hESCs maintained at 20% was plotted against the relative expression of GLUT3 in the same sample. In addition, OCT4 and GLUT3 protein expression was measured in Hues-7 hESCs maintained at 5% oxygen and transfected with either a negative control siRNA or GLUT3 siRNA. This provided more data points to examine any potential correlation between OCT4 and GLUT3 (Fig. 5B). A statistically significant positive correlation was found between GLUT3 and OCT4 expression, using a Spearman rank correlation statistic (Fig. 5B; p = 0.0438; r = 0.6606). As there appeared to be a non-linear, possibly logarithmic, relationship between GLUT3 and OCT4, the relative measures of GLUT3 expression were log transformed. A graph of relative OCT4 expression against the log transformed relative GLUT3 expression was plotted and a significant, positive linear correlation was found (Fig. 5C; p = 0.0185; r = 0.7217).

### PKM2 is localised to the nucleus of hESCs and is regulated by environmental oxygen

**Figure 6:**
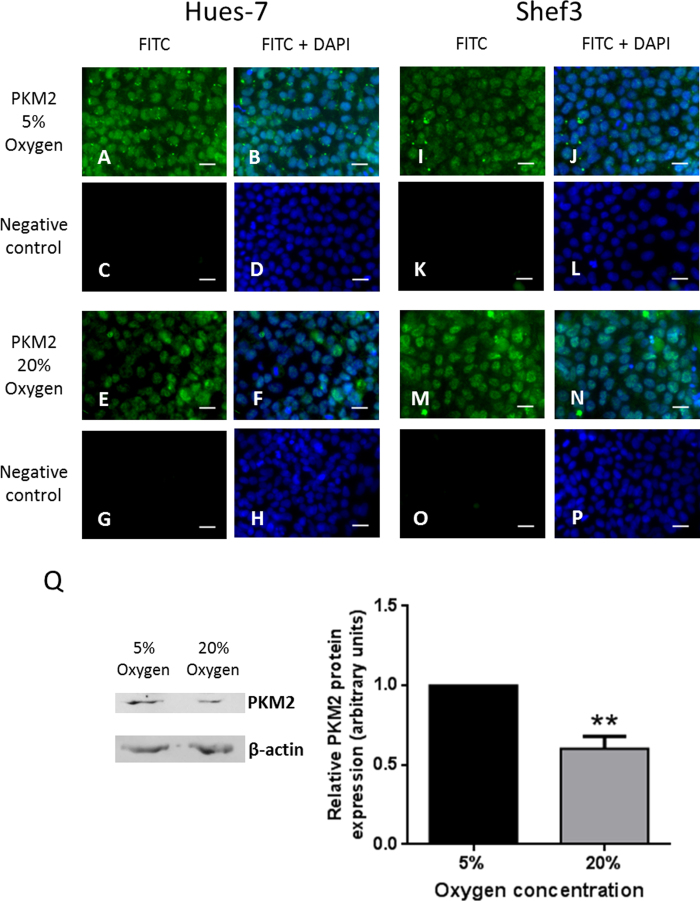
PKM2 expression is localised to the nucleus and is regulated by environmental oxygen in hESCs. Hues-7 and Shef3 hESCs cultured on a MEF feeder layer at either 5% or 20% oxygen were labelled for PKM2, which was localised to the nucleus in both cell-lines at both oxygen concentrations (**A,E,I,M**). FITC-tagged secondary antibodies were used for detection of the primary antibody and for negative, secondary antibody only controls (**C,G,K,O**). DAPI was used to visualise the nuclei (**B,D,F,H,J,L,N,P**). Scale bar = 25 μm. (**Q**) Western blotting was used to quantify PKM2 protein expression in Hues-7 hESCs cultured at either 5% or 20% oxygen using the housekeeping protein β-actin as a loading control. Representative blots are shown for PKM2 and β-actin. Data were normalised to β-actin and to 1 for 5% oxygen. A significantly lower level of expression of PKM2 was found in Hues-7 hESCs maintained at 20%, in comparison with those cultured at 5% oxygen (p = 0.022; n = 4). Bars represent mean ± s.e.m.

PKM2 is an important glycolytic enzyme that catalyses the conversion of phosphoenolpyruvate to pyruvate. Its expression in hESCs was investigated to determine whether it may also have a role in the hypoxic regulation of metabolism. Immunocytochemistry was used to investigate the localisation of PKM2 protein in Hues-7 and Shef3 hESCs maintained at either 5% or 20% oxygen. PKM2 was primarily detected in the nuclei of Hues-7 and Shef3 hESCs maintained at both oxygen concentrations (Fig. 6A,E,I,M).

Western blotting was used to quantify expression of PKM2 in Hues-7 hESCs maintained at either 5% or 20% oxygen. PKM2 expression was significantly lower in hESCs maintained at 20% oxygen compared to those cultured at 5% oxygen (Fig. 6Q; p = 0.022).

### PKM2 regulates OCT4 expression, but does not affect glucose metabolism

**Figure 7:**
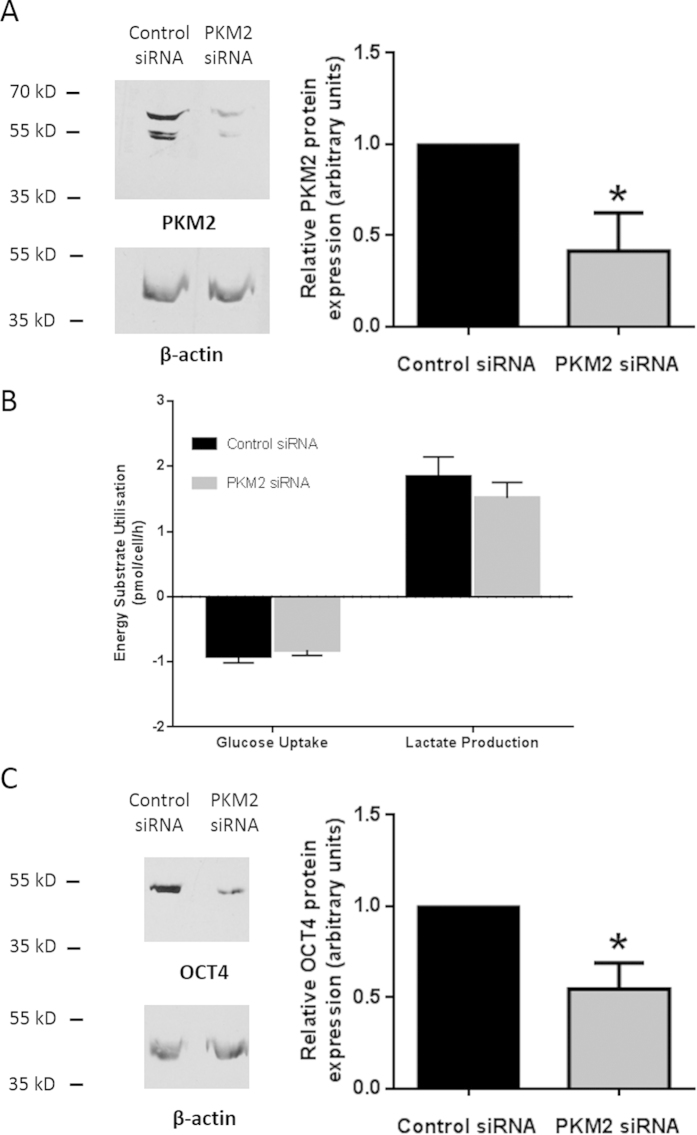
Silencing PKM2 expression in hESCs reduces OCT4 expression, but does not affect glucose uptake or lactate production. Hues-7 hESCs cultured at 5% oxygen were transfected with either a negative control siRNA or siRNA against PKM2. (**A**) Western blotting was used to quantify PKM2 protein expression after transfection. Representative blots are shown for PKM2 and β-actin. Data were normalised to β-actin and to 1 for control siRNA-transfected cells. A significantly lower level of expression of PKM2 was measured after transfection with PKM2 siRNA, in comparison with hESCs transfected with a negative control siRNA (p = 0.0316; n = 4). (**B**) Enzyme-linked assays were used to measure rates of glucose uptake and lactate production after transfection with either a negative control siRNA or PKM2 siRNA. No significant differences between control siRNA and PKM2 siRNA were found for glucose uptake (n = 16 and n = 14, respectively) or lactate production (n = 16 and n = 11, respectively). (**C**) Western blotting was used to quantify OCT4 expression after transfection with negative control siRNA or PKM2 siRNA. Representative blots are shown for OCT4 and β-actin. Data were normalised to β-actin and to 1 for control siRNA-transfected cells. A significantly lower expression of OCT4 was measured after transfection with PKM2 siRNA than after transfection with a negative control siRNA (p = 0.0189; n = 4).

PKM2 was silenced to investigate the potential role of PKM2 in the control of glycolytic metabolism and pluripotency. Hues-7 hESCs maintained at 5% oxygen were transfected with 100 nM PKM2 siRNA and expression of the protein was assessed using Western blotting. Transfection with siRNA against *PKM2* resulted in a significant, approximate 60%, reduction in protein expression compared with Hues-7 hESCs transfected with a negative control siRNA (Fig. 7A; p = 0.0316).

The effect of silencing PKM2 expression on the rates of glucose uptake and lactate production was investigated. No significant difference in glucose consumption or lactate production was found between Hues-7 hESCs transfected with PKM2 siRNA and those transfected with a negative control siRNA (Fig. 7B).

Western blotting was also used to determine whether silencing PKM2 expression has an effect on OCT4 expression. There was a significant reduction in OCT4 expression after transfection with PKM2 siRNA in comparison with Hues-7 hESCs transfected with negative control siRNA (Fig. 7C; p = 0.0189).

## Discussion

Understanding of the mechanisms that regulate hESC maintenance and differentiation has increased significantly over recent years, but energy metabolism has been largely overlooked. This is surprising following the renewed interest in metabolism in other fields, including cancer biology. The biosynthetic and energetic requirements of a cell are supplied by metabolism and vary as the function of a cell is altered during processes such as differentiation or reprogramming^38^,^39^,^40^,^41^. Manipulating metabolism can impede or facilitate these changes in cell identity^36^,^42^,^43^,^44^. Previously, we have identified an association between a reduction in the rate of glycolysis and the expression of pluripotency markers during hESC culture in the absence of FGF2 or with culture at atmospheric oxygen, compared to 5% oxygen^9^. In the current study, we have demonstrated that environmental oxygen regulates glycolytic metabolism and pluripotency through regulation of GLUT3 and PKM2.

Glucose is an important energy source, but how it is transported into hESCs is not known. Glucose uptake in hESCs was previously found to be regulated by environmental oxygen, with a greater rate of consumption in hESCs cultured at 5% oxygen than in those maintained at 20% oxygen^9^. In addition, expression of *GLUT1* mRNA was greater in hESCs cultured under hypoxia and its expression was directly regulated by HIF-2α^9^. However, for transport proteins, control of function can take the form of transcriptional regulation, post-translational modification, or involve the regulation of protein trafficking to the membrane. The presence of the transporter in the cell membrane must be the most important characteristic of the glucose transporter responsible for glucose uptake in hESCs, as regulation of gene expression has no purpose unless the protein product is trafficked to the membrane to perform its function.

Localisation of GLUT transporter expression by immunocytochemistry showed that, in contrast to GLUT1, GLUT3 was consistently present at the membrane of hESCs cultured at both 5% and 20% oxygen (Fig. 1). Using Western blotting a significantly greater expression of GLUT3 was measured in Hues-7 hESCs cultured at 5% oxygen compared with those maintained at 20% oxygen (Fig. 2). This suggests that GLUT3 may be responsible for the previously measured increased rate of glucose consumption in hESCs cultured under hypoxic conditions^9^. These data are consistent with previous observations that GLUT3 plays an important role in glucose uptake in developing mouse embryos^26^. Although the mechanism regulating the increased expression of GLUT3 in hESCs is not known, in choriocarcinoma cells it is mediated by HIF-1α^15^. In hESCs, HIF-1α is only responsible for the initial response to hypoxia, whereas the long-term response is regulated by HIF-2α^10^. Therefore, similar to GLUT1, GLUT3 expression may also be transcriptionally regulated by HIF-2α^9^.

To investigate whether GLUT3 regulates glucose metabolism and pluripotency, siRNA was used to silence GLUT3 expression. A significant, approximate 70%, reduction of protein expression was achieved (Fig. 4A) and importantly, no effect was seen on the level or localisation of expression of GLUT1 (Figs 3 and 4), indicating the specificity of the gene knockdown and lack of compensation by GLUT1. The primary function of glucose transporters is to allow entry of glucose into the cell and silencing expression of GLUT3 led to a significant reduction in the rate of glucose consumption (Fig. 4C). Transfection with siRNA abrogated the hypoxia-induced increase in GLUT3 expression in Hues-7 hESCs and this led to a glucose uptake rate intermediate to the previous measurements of glucose uptake in hESCs at 5% and 20% oxygen^9^. These findings demonstrate that GLUT3 is responsible for glucose uptake in hESCs and suggest that the increased GLUT3 expression observed at 5% oxygen drives the increased rate of glucose consumption in hESCs maintained in hypoxia^9^. A similar decrease in the rate of lactate production was observed after transfection with siRNA against *GLUT3* (Fig. 4C). It is likely that the reduced rate of lactate production is caused by the reduced rate of glucose uptake, as one molecule of glucose is converted into two molecules of lactate through the process of glycolysis. Together, these data suggest that there is a decreased glycolytic flux when GLUT3 expression is silenced.

Silencing GLUT3 expression has also been observed to lead to a significant reduction in OCT4 expression (Fig. 5A). It is most likely that this reduction in pluripotency is a result of the decreased flux through glycolysis caused by silencing GLUT3 expression, as glycolysis is known to be important for maintenance of pluripotency^37^,^43^. This supports the idea that hypoxia promotes pluripotency, in part, through up-regulation of glycolysis, as reduction of the glycolytic rate in hESCs maintained under hypoxic conditions is sufficient to reduce the expression of OCT4. Importantly, a positive correlation between OCT4 and GLUT3 expression was found in Hues-7 hESCs (Fig. 5B). This correlation appeared to be non-linear, so the measurement of GLUT3 expression was log transformed and found to positively correlate with OCT4 expression (Fig. 5C), suggesting that there is a correlation between pluripotency, or at least OCT4 expression, and glycolytic flux such that reducing the rate of glycolysis will impact hESC pluripotency. This effect on pluripotency is more severe as the expression of GLUT3, and presumably the rate of glucose consumption, is reduced further. This is consistent with the previously measured dose-dependent effect on early development, including aberrant brain organogenesis, measured when a morpholino was used to silence the GLUT3 orthologue in zebrafish^45^.

In addition to regulating glucose entry, glycolytic flux may also be regulated through control of glycolytic enzymes. PKM2 is a glycolytic enzyme that is regulated by multiple mechanisms, including allostery, post-translational modifications and control of cellular localisation^46^,^47^,^48^,^49^. However, little is known about the regulation of PKM2 in hESCs. This study investigated the effect of environmental oxygen on PKM2 expression and the role of PKM2 in the regulation of metabolism and pluripotency.

PKM2 was found to localise primarily to the nucleus of Hues-7 and Shef3 hESCs at both 5% and 20% oxygen (Fig. 6). Previously, PKM2 expression has been detected in the nucleus of cancer cells and it has various roles in the regulation of transcription^50^,^51^,^52^,^53^,^54^. However, transcriptional roles of PKM2 have not previously been demonstrated in hESCs. Thus, the localisation of PKM2 in the nucleus of hESCs suggests that it has a function other than as a glycolytic enzyme. If PKM2 expression is nuclear, then it is not clear how the high glycolytic rate is maintained in hESCs. It is possible that there is a lower level of PKM2 expression in the cytoplasm that was undetected by immunocytochemistry or that PKM1 facilitates glycolysis. Another possibility is that a previously described alternative pathway in cancer cells is also present in hESCs and that phosphoenolpyruvate can be converted to pyruvate by acting as a phosphate donor, with the phosphate group transferred onto the glycolytic enzyme phosphoglycerate mutase (PGAM1)^55^. To further investigate the role of PKM2 in the regulation of hESC metabolism and pluripotency under hypoxic conditions, siRNA was used to silence PKM2 expression. This led to an approximate 60% reduction in PKM2 protein expression (Fig. 7A). There was no significant effect of silencing PKM2 expression on glucose uptake or lactate production, suggesting that PKM2 is not required to support a high glycolytic flux in hESCs maintained under hypoxic conditions (Fig. 7B). This suggests that glycolysis is facilitated by PKM1 or the transfer of a phosphate group from phosphoenolpyruvate to PGAM1 rather than by a low level of expression of PKM2 in the cytoplasm of hESCs.

A significantly lower level of expression of PKM2 was observed in Hues-7 hESCs maintained at 20% oxygen in comparison with those cultured at 5% oxygen (Fig. 6Q). As HIF-1α is only involved in the initial response to hypoxia^63^, it is likely that expression of PKM2 is regulated under hypoxia by HIF-2α, in hESCs. Alternatively, it is possible that PKM2 expression is transcriptionally regulated by OCT4, as has been found in mESCs^37^, and the hypoxia-driven increase in OCT4 expression leads to an increased expression of PKM2. If PKM2 primarily functions as a transcriptional regulator in hESCs, then it is likely that hypoxia-driven increases in expression will promote this function.

Interestingly, PKM2 has been demonstrated to interact with HIF-1α to promote transcription of target genes^35^,^54^. It is not known whether PKM2 is able to form a similar interaction with HIF-2α, but it is possible that this interaction could be a major function of PKM2 in hESCs. Another interaction that PKM2 has been found to make, in cancer cells, that could be particularly interesting in the regulation of hESC pluripotency, is with OCT4. However, the function of this interaction is not clear as contradictory results have been presented, with one study demonstrating cooperation^34^ and another suggesting that PKM2 inhibits the function of OCT4^56^. Importantly, silencing expression of PKM2 in Hues-7 hESCs cultured at 5% oxygen resulted in a significant reduction in OCT4 expression (Fig. 7C). This demonstrates that PKM2 regulates OCT4 expression. Hence, rather than having a glycolytic role in hESCs, PKM2 is likely to transcriptionally regulate OCT4. Similarly, it was recently demonstrated that overexpression of Pkm2 in mESCs supported Oct4 expression in the absence of LIF^37^. If PKM2 is able to interact with HIF-2α to promote transactivation of target genes, then it is likely to enhance transcription of OCT4, as OCT4 is a target of HIF-2α in hESCs^63^. In addition, if PKM2 is able to bind to OCT4, and enhance its transcriptional activity, in hESCs, as has been demonstrated in a transformed cell line, this could lead to an increased expression of OCT4 due to the way in which the pluripotency factors act in a self-promoting network^57^,^58^,^59^. Therefore, loss of PKM2 could lead to a reduction in expression of OCT4 by two distinct mechanisms. Further work is required to determine the mechanism by which PKM2 promotes OCT4 expression.

In the current study, we have determined a positive correlation between expression of the proteins GLUT3 and OCT4, suggesting that glucose metabolism regulates pluripotency. In addition, we have demonstrated that PKM2 promotes OCT4 expression. Hypoxia was found to have an effect on the expression of both PKM2 and GLUT3, which highlights two further ways in which culture at a low oxygen tension is beneficial to the maintenance of highly pluripotent hESCs; support of the required high rate of glycolytic flux through the regulation of GLUT3, and the promotion of PKM2 expression, which functions as a transcriptional regulator.

## Methods

### hESC culture

Hues-7 (D. Melton, Howard Hughes Medical Institute/Harvard University)^60^ and Shef3 (supplied by the UK Stem Cell Bank)^61^ hESCs were cultured at 20% oxygen on γ-irradiated mouse embryonic fibroblasts (MEFs; a primary source derived, in institutional facilities following University of Southampton ethical review committee approval and in accordance with UK Home Office regulations) in Knockout DMEM (Invitrogen) that was supplemented with 15% knockout serum replacement (Life Technologies), 1% non-essential amino acids (Life Technologies), 1% penicillin/streptomycin (Life Technologies), 1% L-glutamax (Life Technologies), 50 μM β-mercaptoethanol (Sigma) and 10 ng/ml bFGF (Peprotech). hESCs were then transferred to Matrigel (BD Biosciences) coated plates and cultured in MEF-conditioned supplemented Knockout DMEM at both 20% and 5% oxygen. hESCs were maintained for a minimum of 3 passages on Matrigel at both oxygen tensions before use.

### Immunocytochemistry

hESCs were fixed in 4% paraformaldehyde for 15 minutes before being blocked for 1 hour in 3% donkey serum in PBS. Cells were permeabilised using 1% Triton X-100. Cells were incubated with primary antibodies, diluted in 3% donkey serum and, if intracellular antigens, in 1% Triton X-100 overnight at 4 °C. Primary antibodies used were GLUT1 (Abcam) 1:250, GLUT3 (Abcam) 1:100, OCT4 (Santa Cruz) 1:100, and PKM2 (Novus) 1:100. Cells were then incubated with the secondary antibody for 2 hours at room temperature. The secondary antibody used for GLUT1, GLUT3, and PKM2 was goat anti-rabbit Alexa 488 (Life Technologies) 1:800 and for OCT4 the secondary antibody was goat anti-mouse IgG FITC (Sigma) 1:100. Cells were mounted in Vectashield with DAPI (Vector Laboratories).

### Western blotting

Hues-7 hESCs were lysed in ice cold radioimmunoprecipitation assay (RIPA) buffer (Sigma). Samples were incubated for 20 minutes on ice before sonicating for 30 seconds. 50 μg protein was resolved on a 12% SDS bisacrylamide gel before being transferred to a nitrocellulose membrane and blocked with 5% milk in PBS containing 0.1% Tween for 1 hour at room temperature. The membrane was incubated with primary antibody diluted in the blocking buffer overnight at 4 °C. Primary antibodies against OCT4 (Santa Cruz) 1:1000, GLUT1 (Abcam) 1:1000, GLUT3 (Abcam) 1:3000, and PKM2 (Novus) 1:1000 were used. Membranes were then washed and then a horseradish peroxidase-conjugated anti-mouse antibody (GE Lifesciences), 1:100,000, was used for detection of OCT4 and a horseradish peroxidase-conjugated anti-rabbit antibody was used for detection of GLUT1, GLUT3, and PKM2 (GE Lifesciences), 1:50,000. Amersham enhanced chemiluminescence Western blotting detection reagents (GE Lifesciences) were used along with film development for band detection. Protein expression was quantified relative to β-actin (mouse anti-β-actin horseradish peroxidase-conjugated antibody (Sigma) 1:50,000).

### Transfection with siRNA

siRNA was used to silence expression of GLUT3 and PKM2 in Hues-7 hESCs cultured at 5% oxygen on Matrigel-coated plates. hESCs were transfected on the first day after passaging, before a standard medium change 24 hours later. Experiments were performed, or protein was collected, 48 hours after transfection. To silence GLUT3 expression, 50 nM of a previously validated siRNA (Ambion) was used with the transfection reagent INTERFERin (Polyplus). To silence PKM2 expression, 100 nM of a previously described PKM2 specific siRNA (Sense strand: 5′ CCAUAAUCGUCCUCACCAAUU 3′; Thermo Scientific)^32^ was required, with the transfection reagent HiPerFect (Qiagen). Allstars control siRNA (Qiagen) was transfected into hESCs alongside GLUT3 siRNA and PKM2 siRNA experiments as a negative control.

### Measurement of hESC carbohydrate utilisation

Metabolic analysis was performed with hESCs cultured in 12-well Matrigel-coated plates. 6 wells, containing cells cultured on Matrigel, were used for analysis and 4 cell-free wells were used as controls. On the third day after passaging, the culture medium was removed and replaced with 500 μl of a defined metabolic medium^62^, containing amino acids, 470 μM pyruvate, 1 mM glucose, and 5 mM lactate, for 30 minutes to allow acclimatisation of hESCs to the medium. The medium was then replaced with 300–500 μl of the defined medium for 1.5–3.5 hours. After this incubation, 200 μl was removed and stored at −80 °C and a haemocytometer was used to count the number of cells in each well. Enzyme-linked assays were used to measure the concentration of glucose and lactate in the spent medium to allow calculation of rates of glucose consumption or lactate production in pmol/cell/hour^9^. A Fluostar Optima microplate reader (BMG Labtech) was used to measure fluorescence of NADPH after a glucose assay and NADH after a lactate assay.

### Statistical analysis

D’Agostino & Pearson omnibus normality tests were performed on datasets to determine whether parametric or non-parametric tests were appropriate. Depending on the results, either Mann-Whitney U or unpaired Student’s t-tests were performed to compare protein expression or rates of nutrient uptake or production between culture conditions. Protein expression was normalised to β-actin and to 1 for cells cultured at 5% oxygen or cells transfected with negative control siRNA. To analyse the correlation between GLUT3 and OCT4 expression, a Spearman rank correlation test was used. In all cases a value for p < 0.05 was taken to indicate statistical significance. All data represent a minimum of 3 independent experiments and are presented as mean ± s.e.m.

## Additional Information

**How to cite this article**: Christensen, D. R. *et al.* GLUT3 and PKM2 regulate OCT4 expression and support the hypoxic culture of human embryonic stem cells. *Sci. Rep.* **5**, 17500; doi: 10.1038/srep17500 (2015).
